# Controlling Variables in Molecular Gel Science: How Can We Improve the State of the Art?

**DOI:** 10.3390/gels4020025

**Published:** 2018-03-22

**Authors:** Richard G. Weiss

**Affiliations:** Department of Chemistry and Institute for Soft Matter Synthesis and Metrology, Georgetown University, Washington, DC 20057-1227, USA; weissr@georgetown.edu

**Keywords:** gel formation protocols, component purity, gel history, rheometry, microscopy, diffraction

## Abstract

By design, no references are included in this article. It is intended to be a series of recommendations in which the focus is on lab practices for investigating substances rather than on the substances being investigated. Thus, it discusses some specific areas of concern identified by the author. Other scientists are encouraged to add to or amend the contents. This article should be read as a “living” document, like a blog in which many gel scientists work, over time, to achieve a consensus about reporting everything from acronyms and definitions to procedures and methods. For those entering the field and seeking compendia on the subject, the author suggests “Googling” the words “molecular gels” or “molecular gels books”.

*Where are we and where are we going?* The last 2–3 decades have witnessed remarkable advances in fundamental and applied aspects of research on molecular gels (i.e., those comprised of a liquid and a “small” molecule in which the gelator network is held together by physical intermolecular interactions). They differ from polymer gels in which the networks rely on chemical or physical interactions among chains, but the chains, themselves, are held together by covalent bonds. Here, the author offers a polemic (perhaps better, “an exhortation”) addressed to those (like him) who are interested in molecular gels or intend to enter the field: We should strive to be much more rigorous in reporting the procedures we employ to make and characterize molecular gels. In fact, many of the concerns raised here apply to polymer gels as well. 

*Avoiding a key question: “What is a gel?”* An important aspect of this discussion is finding a common definition for *what is a gel*. One person’s gel may be another person’s sol, high viscosity polymeric dispersion, or even precipitate. For that reason, the criteria for designating a sample as a gel (or another phase) should be described within the context of each study being reported. Although this issue is discussed more in Section 5, it is not settled. We are still trying to find a simple, universally acceptable answer to the question, *“What is a gel?”* Does one exist? The author avoids this question here out of a mixture of cowardice and ignorance.

*Mea culpa*. In some reports in the literature on both molecular and polymer gels, the lack of adequately detailed information has resulted in an inability of others to reproduce some of the data. The author is as guilty as anyone else for reporting only some of the experimental details necessary for others to reproduce some of his observations and data. As a result, development of the field of gel research has suffered. The author will try to be more careful in the future, and he asks his colleagues to do the same; all of us will benefit! As mentioned above, no references are included within this document; if this were a finger-pointing exercise, the author fears that each hand would need hundreds of digits, and many would be directed toward him!

A fundamental question for data associated with gels, as with any other set of data, is, “Can the results be reproduced by others in other labs?” A basic test of the validity of a method of sample preparation and experimental procedures used is reproducibility. Also, if the data cannot be/have not been reproduced quantitatively (including with error limits), their value should be treated with doubt. 

## 1. Liquid and Gelator Purity

Although it may seem obvious, the materials used to form a gel must be as pure as possible. This includes specification of the enantiomeric and geometric content of chiral gelators and those capable of existing in *cis-trans* or other isomeric forms. Their source/supplier/grade/purity should be specified. If the liquid has been treated in some way by drying or distilling, for example, the procedures should be noted in detail. Similarly, if the gelator has been synthesized, sufficient characterization data to delineate its structure and purity should be included within the Experimental section of the article in which the results are reported. For example, if traces of the solvent from which the gelator has been isolated or crystallized remain, they may affect the eventual gelating characteristics. 

In fact, there are examples in which different morphs of a gelator (produced from crystallization in different solvents; many gelators are polymorhous) result in different gelating abilities. In principle, this should not happen if the gel is formed by cooling a sol phase (See Section 2) that has been heated to a sufficiently high temperature for a sufficiently long time. In practice, it is very difficult to know experimentally what are “sufficient” conditions to ensure that the gelator molecules have been transformed in a sol to completely isolated molecules (i.e., *without any vestige of small aggregates*). Only under those conditions can one be sure that the gelator does not retain some memory of its prior morphology. When gels are made by other protocols that do not involve the complete dissolution of the gelator molecules (See Section 3 and Section 4), gelator history becomes an even more important issue. 

## 2. If Gels Are Being Formed by Cooling Sols to Below Their Gelation Temperatures (Tg)

The initial temperature of the sol and its rate of cooling should be specified (i.e., xxx deg/(unit of time)). Frequently, neither of these is reported although it is known that some molecular gelators adopt different morphs depending, especially, on the rate of cooling. In addition, if the liquid and gelator are not heated initially to a sufficiently high temperature and for a sufficiently long period of time, different degrees of aggregation, which influence how the gelator networks form, may be present before cooling is commenced. Thus, the sol may be predisposed to form a different type of network than one kept at another temperature above Tg.

1. Especially, Tg is found to depend acutely on gelator concentrations, especially when the latter is low. There, very small variations in gelator concentration effect large changes in Tg. The origin of these changes may be in a factor as mundane as small weighing errors or in modifications of how a sol is being cooled. For that reason, it is advisable to construct Tg-gelator concentration phase diagrams and, where possible, employ gelator concentrations in the “plateau region”, where Tg is relatively constant.
2. “Slow cooling” and “fast cooling”, descriptions frequently employed, for two protocols are meaningless unless defined in much more detail. Especially in the case of “fast cooling”, even when the rate is specified, there can be problems with reproducibility because the internal temperature of the sample may be sensitive to the heat capacity of the liquid in the sol and, thus, the size of the sample. In addition, the actual rate of cooling will change if a sample is placed in a larger bath that is allowed to cool as well to room temperature (or below) because the rate will slow as the differential between the bath and final temperature becomes smaller. Of course, this complication can be avoided if the bath temperature is cooled at a programmed rate by a Peltier or similar device.
3. Another method involves cooling the sol as rapidly as possible to a set temperature below Tg and incubating there until the gel is formed. Again, there is a potential problem—the rate of cooling to the incubation temperature. This rate can be controlled as noted above.

In some cases, it can be demonstrated that the rate or protocol for cooling has no discernible effect on the structure and viscoelastic properties of the gel. When that is so, it should be stated, along with the evidence supporting the conclusion.

## 3. If Gels Are Being Formed from Sols by Ultrasonic Treatments

As in (Section 2), several parameters must be specified in order for others to be able to reproduce the protocol. Those parameters include the initial temperature of the sol and how it was prepared (Section 2.1). Of course, the time of application and the frequency of the ultrasound, as well as the total energy deposited, must be specified. Because the profile for signal attenuation is exceedingly important, the location of the ultrasonic source with respect to the sol (i.e., inside the sample and in its center or external to it) must be specified. If the radiation is applied unidirectionally, the energy deposition throughout the sample will be attenuated at longer distances. Also, any temperature changes that occur during the energy deposition should be reported. 

## 4. If Gels Are Being Formed or Converted to Sols by Addition of Additives Such as Small Amounts of an Acid or Base (to Change the pH), Metal Ions, and Enzymes, or under the Influence of UV-Vis Radiation, Electrochemical Potentials, etc.

Each of these perturbations requires a different form of documentation to ensure the ability of others to reproduce a result. The method of addition of additives, including the materials for changing pH should be specified. At what stage and in what way were they added—to sols, to already formed gels, the temperature of the sample before addition, the rate of addition, etc.? 

The last factor listed above may become very consequential if the rate of gelation is faster than the rate of addition. Under those circumstances, it is likely that inhomogeneous gels will result; the rate of mixing the added component should be must faster than the rate of gelation and, in any case, that rate and the method of mixing should be specified. 

### 4.1. Addition of an Acid or Base to Effect pH Change

If the added base or acid is delivered neat or in a solvent different from that in the gel, its effect on the macroscopic properties of the liquid portion should be considered. For example, the concentration of the acid or base will determine the volume of the delivery liquid that must be added to attain the desired pH. Also, as noted above, the manner in which the gelator nucleates and forms a network may be altered by whether the acid or base is added dropwise with stirring or all at once at the top surface of the sol/gel. If added all at once, there will be concentration gradients not only for the acid or base, but also for the other species with which they interact. Those species may include products needed for gelation or for its loss if a chemical reaction other than salt formation is occurring. 

### 4.2. Addition of Metal Ions

The same considerations as described in Section 4.1 apply to the addition of metal ions and even enzymes. In the latter case, the activity of the enzyme should be mentioned, as well. In the former case, it is necessary to determine whether the presence of the metal ions is acting to oxidize or reduce the gelator (see Section 4.4), to change the ionic strength of the liquid, or to modify the type of electrostatic interactions within ionic gelators.

### 4.3. UV-Vis and Ionizing Radiation

To date, most forms of radiation used to change the properties of a gel have been in the form of UV-vis. However, ionizing radiation has been employed in some systems and it may become more popular in the future. 

In addition to specifying the wavelength(s) of UV-vis radiation impinging on a sol or gel, the rate of photon absorption (i.e., watts absorbed or energy absorbed per unit of time) and the dose (i.e., the total amount of energy absorbed by a unit mass of a sol or gel). Application of these definitions requires knowledge of the surface area being irradiated by and the thickness of the sample in order to know whether the radiation is being deposited homogeneously throughout or whether there is a spatial gradient for photon deposition. Of course, the effective nature and intensity of the radiation will depend on the lamp profile and the presence or absence of filters that can include the material of the sample container (e.g., glass or quartz) or even a water bath, and the temperature of the sample during irradiation should be controlled if possible and monitored if not. Because the number of photo-induced events in each volume segment of the sample will depend on the profile for photon absorption throughout the sample, being able to reproduce the effect of irradiating a sol or gel can be a very difficult chore. In fact, as long as the photo-induced events persist for periods much longer than the period of irradiation, the rate of photon deposition will not be an important consideration. However, the local concentrations of photo-induced species will continue to be. 

### 4.4. Electrochemical Redox Processes

If gels and sols are being interconverted electrochemically, the nature and size of the electrodes, the potential applied and the time of its application, the concentration of any added electrolytes, the atmosphere above the sample, and the degree of reversibility must be specified. Because diffusion of objects to and from the electrodes will change as the objects within the gelator network are formed or lost, it may be difficult to reach equilibrated phases. Thus, it is necessary to determine the concentrations of the oxidized and reduced species after application of the voltage for specific periods. The electrochemical characteristics of the system should also be conducted in a solvent in which no gel is formed as a means to compare the redox properties of the gelator molecules in the absence of their nucleation.

## 5. Preliminary Determination (Screening) of What Is a Gel (Visually)

1. The first indicator of the nature of the nature of a material should be visual. When reporting the appearance, the thickness of the sample should be noted because a thin sample may appear to be translucent while a thicker one is opaque. Furthermore, the appearance of the gels should be documented with optical micrographs and they should be recorded periodically in normal room light if changes are observed over time; see Section 8. A transparent or translucent sample that does not flow when inverted (i.e., resists the force of gravity) or has a rheologically-determined *G*″/*G*′ ratio (i.e., a tan*δ* value near or lower than 0.1; see Section 10) can be classified preliminarily as a gel. However, the period during which the sample has been kept in an inverted position should be specified—one sample may not flow after one minute but significantly after one hour, even though it may or may not be a gel.
  Also, appearance is important! Those materials that are cloudy or opaque may be gels or finely separated and very weakly interacting crystalline objects. They. Like those that resist flow after long periods, require additional studies to provide definitive classifications.
2. As noted in Section 6, the outward appearance of a sample may depend on its size (as limited by the dimensions of the container). Thus, the visual appearance of a sample may not provide an accurate assessment of whether a gel, *as defined rheologically*, can be formed. In fact, some gels are not formed when the volume is too large (due to issues related to gravity) and others when it is too small (due to issues related to the size of the objects necessary to sustain a network). The latter issue is endemic to many of the techniques employed to characterize gels (e.g., rheometry, UV-vis and IR spectroscopic measurements, optical microscopy, X-ray diffraction and small angle neutron scattering). Also, some samples are sensitive to the material or surface roughness (e.g., from scratches) of the walls of the container because they may catalyze or inhibit nucleation and growth of the objects within a gel network. Not only may they alter the kinetics of network formation, they may also change the nature of the objects within the network. Thus, because the container surfaces (e.g., glass, Teflon, other plastics, whether surface derivatized, etc.) can contribute in important ways to the characteristics of a gel, and even whether it will be formed, the nature of the surfaces directly in contact with the gel precursor should be specified.
3. Related to these issues is another—the presence or absence of dust particles or gas bubbles in the liquid component that can, again, catalyze or alter nucleation and growth of the objects of a gelator. Precipitates rather than gels (or gels rather than precipitates!) may result from the interactions of dust particles or initially dissolved gases with gelator molecules at the nucleation and growth stages of aggregation. For that reason, it is especially important to prepare sol phases that are dust free.

## 6. Gel History and Structure

1. The structure of the gel network and the viscoelastic properties of the gel may be very dependent on the length of time since the gel was first made from its sol and how it was made from the sol. Over time, many gels undergo structural and viscoelastic changes, and eventually undergo complete changes into a solid and a liquid; molecular gels are not thermodynamically stable. For that reason, the age of a gel and its mode of storage, especially temperature—the temperature at which a gel is stored may prolong or shorten its lifetime—should be specified. Some of the changes may relate to sample size, the container shape and its dimensions, and the effects of gravity. Two important aging phenomena are syneresis (i.e., the expulsion of the liquid component from a gel, leading to separate layers and contraction of the volume in which the gelator network resides) and Ostwald ripening (i.e., the thermodynamically driven process that converts smaller objects in a gel network into larger ones). The time profile for each depends on the liquid, gelator structure and concentration, the storage conditions (e.g., temperature and whether they have been agitated mechanically).
2. There are other, more mundane (but equally important) factors that must be taken into account related to the storage container. It must be sealed carefully, to avoid: (a) evaporation of the liquid component, leading to an increase in the gelator concentration; (b) entry of moisture from the atmosphere that can be an important problem if one or both of the gel components is hydrophilic.
3. When using the spectral properties of a gelator to ferret out details about intermolecular interactions, the proper states and aggregates should be compared. For example, infrared spectra of (dried and possibly morphologically distinct) xerogels should not be analyzed as models for gel networks. However, spectra recorded on samples containing gelator and liquid above and below Tg may be enlightening. A problem with these comparisons is the need to subtract the absorption bands of the solvent from those of the solute and to separate the spectral features of the gelator within the aggregate network from those dissolved within the liquid component (i.e., the critical gelator concentration). Under the best of circumstances, such subtractions can be somewhat imprecise; at low gelator concentrations, they can be very imprecise!
  When gels are opaque or only partially transparent, the intensity and resolution of both FT-IR and UV-vis absorption spectra can be affected by reflections at the interfaces between the objects within the gel network and the liquid unless (in rare cases) the two are index of refraction matched, This complication, resulting in a loss of quantifiable data, can be overcome by placing samples in diffuse reflectance apparatuses, such as integrating spheres. Although this approach allows quantifiable absorption spectra to be recorded, care must be taken to ensure that (as before) liquid is not lost by evaporation and that proper control spectra of the sample holder cell and liquid are recorded under the same conditions as the gel.
4. Even when a gel is made under well controlled conditions, how it is treated during variable temperature analyses may provide data whose attribution is questionable. An example is data obtained from calorimetric measurements. The enthalpies of transitions from differential scanning calorimetry (DSC) in hermetically sealed sample containers may depend on the rate of heating because some of the liquid may vaporize and the peaks may be small if the concentrations are like those of the dilute gels. More importantly, the Tg values and heats obtained from reheating the gel phases of the same samples may not be reflective of the values intrinsic to the original gel if the rates of cooling employed to make the sample differ from those used in the DSC experiments. For this reason, it is not uncommon for the values obtained on second heating of a sample to differ from those during the first heating.

## 7. SEM/TEM Sample Preparation Procedures

Frequently, the methods by which SEM and TEM samples have been prepared are very poorly described in publications. As a result, it is difficult to reproduce with confidence the images or to be able to discern the link between the images and their precursor gels. Thus, all of the factors noted in Section 1, Section 2, Section 3, Section 4, Section 5 and Section 6 should be controlled in addition to several others: the Initial concentration of the gelator in the gel, the substrate for the sample (e.g., a Cu grid and its mesh size for TEM or the type of conducting tape for SEM), details of the procedures for removing the solvent (i.e., if removed under vacuum, temperature and pressure and time that the conditions were applied or if dabbed with a tissue), and the nature of any metal/carbon coatings and how they were applied. Alternatively, some samples are “freeze-dried”, a method that reduces the possibility that the original gel structure is modified significantly. Regardless, if one of the methods above is being employed, some evidence that the xerogel being imaged is like the actual gel network should be provided. 

Sample preparation techniques associated with obtaining cryo-SEM and cryo-TEM imagines involve flash freezing of the gel samples. They are the best methods for minimizing the probability that the preparation procedure will produce a change in the packing of gelator molecules within a fiber or within the network. However, not all gel scientists have access to the cryo equipment. Even when a cryo method is employed, information concerning the liquid used for freezing, how the sample was prepared prior to plunging it into the liquid, and how the frozen sample was transferred to the microscope should be specified. Also, for instrumental reasons, films for cryo-TEM must to be very thin (<300 nm). As a result, they can provide valuable structural information about fiber morphology, but not about the network in which the fibers reside.

If a stain was applied, the details of its composition and contact time, and how excess solution was removed should be specified because the stain, itself, is capable in some cases of modifying the structural properties of the fibers. Also, the energy of the electrons and the duration of exposure to them by the sample should be mentioned as a means to allow the reader to assess the possibility that the sample was damaged (“burned”) at the moment when the image was recorded. Finally, it should be made clear whether the images published are representative of the bulk: the best photos should be reserved for family albums; representative ones should appear in published articles! 

Many of the same procedural requirements and caveats apply to experiments employing AFM techniques to image gels. A partial list includes the initial concentration of the sol or gel, how it was deposited on the substrate and the nature of that substrate (e.g., mica, silica, etc), and how the sample was dried if it was converted to a xerogel. Other factors, such as the tip type, whether a scanning or tapping mode was employed, how the samples were protected from surface defects and dust during sample preparation (and after!), and how the raw data were processed (e.g., details about the Fourier filtering) must be detailed. In fact, very few AFM images of actual gels have been reported. The vast majority are xerogels, prepared by drying sols or gels and, therefore, susceptible to surface effects on the substrate during nucleation and growth as well as aggregation or crystallization of objects as the liquid is removed. 

## 8. Optical Micrographs

Some of the more important variables and conditions associated with optical micrographs are discussed below. As before, the procedure for sample preparation (i.e., its history) should be specified. Furthermore, how an aliquot is placed on the microscope should be mentioned. That is: whether a portion of a preformed gel was placed on a glass slide and then another was placed on top of it; whether such a sample was heated to above it gel melting temperature and then returned to its gel state before the optical micrographs were recorded. Other important factors include how the loss of the liquid component was avoided if the gel was heated or if the liquid is very volatile at ambient temperatures. The actual magnification of the image in a publication is insufficient to determine the size of features because a journal may increase or decrease the dimensions of the submitted image. To avoid any ambiguity, a distance scale should be embedded in the figure. Finally, if polarizers or wave plates were employed on the microscope, their specific nature and how they were used should be mentioned. For example, were the polarizers at 90° with respect to each other?

## 9. Nuclear Magnetic Resonance Spectra

The aggregation of gelator molecules intrinsic to gels and many sols limits the information that can be gleaned from normal solution-phase spectra. The very short relaxation times of the aggregates makes their nuclei ‘invisible’; only the gelator molecules remaining dissolved (i.e., the critic gelator concentration) will be detected, although some aggregated species may be seen if exchange rates between the aggregates and single molecules are rapid. 

Although the signals from the aggregates can be recorded successfully using CP-MAS techniques, they require very rapid spinning that may destroy the gel due to centripetal forces. Techniques to overcome even this complication, using micro-bore sample containers, are being developed. Their small size does necessitate the averaging of many FIDs because the gelator molecules within the networks is only a small fraction of the total sample volume. Also, the samples should be examined immediately at the end of data collection to ascertain whether the gel phase has survived. Of course, the data are not usable if the mechanical force led to loss of the gel phase, regardless of the point at which the loss occurred.

Finally, most NMR experiments require a deuterated liquid whereas, most other studies on gels are performed with their protonated analogues. Although the extent to which H-bonding, pH, and even van der Waals forces change in deuterated analogues is expected to be small, it should be kept in mind that protonated and deuterated gels may behave somewhat differently. 

## 10. Oscillatory Rheology

1. As above, the history of the sample and how it was placed between the rheometer plates should be noted. Also, the procedures preceding the recording of data must be specified. They include the final distance between the plates, whether plate-plate or cone-plate tools were used, the metal from which they were made and their diameter, and, the cone angle in the latter case. No oscillatory measurements should be made until the force between the upper and lower plates becomes zero. Then, it should be specified how a linear viscoelastic region was found and at each temperature employed; frequency sweeps should be conducted at strains within the linear viscoelastic region. Even whether frequencies were scanned from higher to lower or lower to higher values should be mentioned. Reproducibility, a frequent problem in rheology, is addressed most easily by demonstrating that different aliquots give very similar results.
2. A potential problem is the creation of micro (or larger) bubbles from outgassing as a sample is being heated or as the plates are brought closer to each other during the loading procedure, because they can have an enormous effect on the ability to reproduce instrumental values. For that and other reasons, measurements should be repeated.
3. Finally, it is imperative that the liquid component not evaporate during the period of measurements. Evaporation, when it occurs, is most prevalent near the edges of the plates. That is the region where the torque is greatest and, thus, where the absence of liquid or presence of concentration gradients have the greatest influence on the measurements. “Solvent tents”, solvent-saturated tissues, and even inert oils placed around the circumference of the two plates (to make an impenetrable barrier) have been used to avoid the problem of liquid evaporation. A practical method to demonstrate the reproducibility of data obtained over time from the same aliquot is to show that the *G*’ and *G*” moduli from a strain sweep match those of the frequency sweep within limits shown by error bars.

## 11. Diffraction Methods

1. Frequently, the distinction between X-ray data for a gel and a xerogel is blurred. The diffraction from actual gels is much more difficult to record because of the large amorphous signal(s) from the liquid components that represent more than 90% of the total volume usually. As a result, very long times are necessary to obtain diffraction patterns with sufficient signal-to-noise ratios that allow the liquid component to be subtracted partially and imperfectly. To do so, the data set for the liquid must be subtracted from that of the gel, leaving primarily small peaks from the solid gelator network. A problem that arises with this technique is that the portion of the liquid in a gel sample that interacts at the liquid-gelator network interface may have different diffraction characteristics than the bulk liquid. Thus, there is sometimes a great deal of empirical data treatment to locate the actual peaks from the gelator network.
  An alternative is to remove the liquid, creating a xerogel. The increased aggregation that accompanies solvent removal and the possible changes packing arrangement in gelator networks that can accompany removal of the liquid make it difficult to correlate the diffraction patterns of the gel and xerogel; questions arise always about the validity of associating packing data from a xerogel with those of the gel. Is a xerogel that exhibits the characteristics of a crystalline material a good representation of the network in the gel, which may not be crystalline?
2. A third alternative is to use a synchrotron source to record the diffraction pattern of a gelator within a gel. Because of the extreme intensity of the synchrotron radiation sources, gels must be exposed for very short times. However, collecting data on different small aliquots usually suffices to produce excellent data sets. Fortunately, small, intense radiation sources are being developed that may make possible synchrotron-like data quality to be obtained in small laboratory settings.
3. Another “indirect method” technique, one whose successful use depends on careful fitting of the data to various scattering models, is small angle neutron scattering (SANS). The data from this technique complement those from X-ray diffraction. As with X-ray diffraction, it is imperative that the samples for SANS be prepared carefully and that their history be recorded fully. In both techniques, a subtle consideration is the sizes of the objects within the sample. The sample holders are usually thin, and, as noted in Section 5.2, if their dimensions are smaller than those of the objects within the gel network, data may be recorded on samples that are not gels; the samples may consist of sols or other phases. Thus, it is important to ascertain that the samples within geometrically constrained vessels are really gels.

Many SANS experiments require partially or fully deuterated liquids. As in NMR spectroscopy (see Section 9), the structure and behavior of gels may be affected somewhat by substituting a deuterated liquid for a protonated analogue. Thus, the extent of those changes should be ascertained.

*Where to from here?* In the Abstract of this manuscript, the author wrote, “This document is intended to be “living”, like a blog in which many gel scientists work, over time, to achieve a consensus about reporting everything from acronyms and definitions to procedures and methods.” He ends by reiterating that desire. Thus, he appeals to all interested in this area of science to emend and amend this document as future developments dictate or if there is disagreement about the statements within it. Partially because of the issues raised here, “molecular gels” has no paradigm. In fact, it lacks a general model for designing and predicting the properties of new molecular gels. The development of one is a supremely important challenge for the future, and the creation of one will depend to some extent on the universal adoption by the community of terms and guidelines!

More practically, many of the issues raised in this manuscript have been known to scientists working in the field of molecular gels for many years. Most of us have ignored some or all of them for reasons of expediency. However, the development of the field has progressed to the stage where it is not possible to continue to do so. If the science of molecular gels is to advance to the next stage, all of us must take responsibility for ensuring that our data are quantified appropriately and our systems are comprehensively described. The ultimate test is whether others reading our scientific reports have the information necessary to reproduce our results both qualitatively and quantitatively. What are the consequences if we fail to do provide that information? There will be very limited advancement in understanding two fundamental aspects of molecular gels:

(1) Why, when, and how do they form?
(2) How can their properties be predicted a priori?

In the opinion of the author, answering these questions is the principal challenge for those working in the field and for those entering it.

